# One-step functionalization of gold nanorods with N-heterocyclic carbene ligands†

**DOI:** 10.1039/d5ra00754b

**Published:** 2025-02-14

**Authors:** Nathaniel L. Dominique, Phattananawee Nalaoh, David M. Jenkins, Richard Vaia, Kyoungweon Park, Jon P. Camden

**Affiliations:** a Department of Chemistry and Biochemistry, University of Notre Dame Notre Dame Indiana 46556 USA jcamden3@nd.edu; b Department of Chemistry, University of Tennessee, Knoxville Knoxville Tennessee 37996 USA; c Materials and Manufacturing Directorate, Air Force Research Laboratory Wright-Patterson AFB Ohio 45433-7702 USA kyoungweon.park.1.ctr@us.af.mil; d Bluehalo Dayton Ohio 45432 USA

## Abstract

Here, we present a one-step approach to append N-heterocyclic carbenes (NHCs) to gold nanorods. The nanorods are treated with NHC gold or silver complexes in a mixture of water and dichloromethane. Surface-enhanced Raman spectroscopy and mass spectrometry characterization reveals that this procedure results in a ligand transfer yielding chemisorbed NHCs.

## Abstract 2

Direct addition of N-heterocyclic carbene silver or gold complexes to CTAC coated gold nanorods yields chemisorbed ligands *via* a facile, one-step procedure.

## Graphical abstract

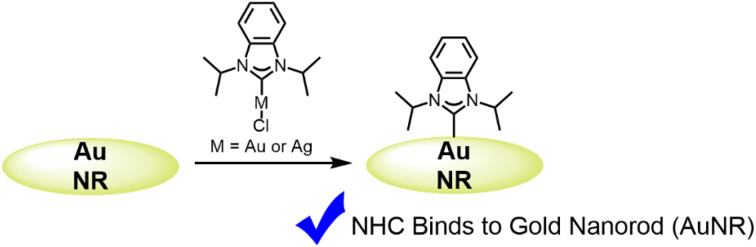

Despite the swift adoption of N-heterocyclic carbene (NHC) technology for nanoparticle research^1–19^ routes to functionalize anisotropic nanomaterials with NHCs remain almost entirely unexplored. For example, gold nanorods are extraordinarily useful for photothermal therapy,^20–22^ near-IR optics,^23^ and displays,^24,25^ but there is only one established method to functionalize gold nanorods with NHC ligands. This approach from Johnson and co-workers^26^ involves a multistep ligand exchange reaction using a bidentate NHC/thiol ligand precursor that must be photoactivated and then chemically reduced to bind to cetyltrimethylammonium bromide (CTAB) coated gold nanorods (Fig. 1A). Although the resulting NHC nanorods are robust for high-performance photothermal therapy,^26^ more facile procedures are necessary to facilitate the widespread adoption of NHC technology.

**Fig. 1:**
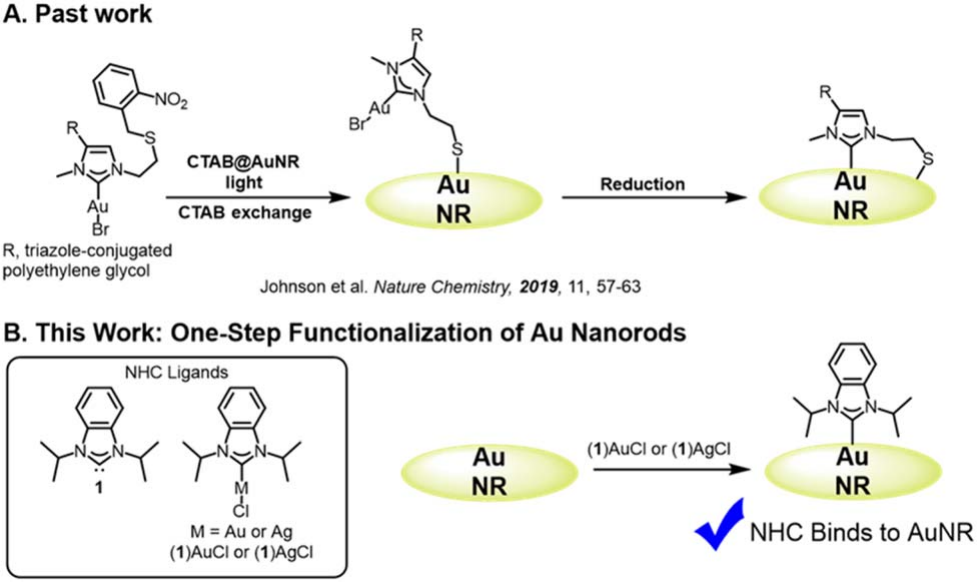
Functionalization of Gold Nanorods (AuNRs) with N-Heterocyclic Carbene (NHC) ligands. The established approach to functionalize gold nanorods relies on a bidentate ligand that must be photoactivated and chemically reduced ((A), ref. 26). This work deploys (1)AuCl and (1)AgCl complexes to append NHC ligands to gold nanorods using a facile, one-step approach (B).

While the thiolate–NHC ligand used by Johnson and co-workers^26^ was necessary to penetrate the dense CTAB bilayer, we hypothesized that standard NHC metal complexes may be able to penetrate a more permeable bilayer. To this end, we prepared gold nanorods coated in cetyltrimethylammonium chloride (CTAC), which is known to bind less strongly to the nanorod surface than CTAB and creates patches of bilayer.^27^

In this report, we demonstrate that NHCs can be appended to CTAC coated gold nanorods using a modified top-down method^28^ starting from NHC gold(i) and silver(i) complexes (Fig. 1B). NHC treated nanorods were characterized using surface-enhanced Raman spectroscopy (SERS) and laser desorption/ionization mass spectrometry (LDI-MS), and our results were benchmarked to an established NHC–gold nanoparticle system. The treatment of gold nanorods with NHC complexes yields SERS and LDI-MS signatures in excellent agreement with a chemisorbed NHC on gold, illustrating that CTAC coated gold nanorods can be directly functionalized with NHC ligands using a facile one-step procedure.

To explore the gold nanorod functionalization process, we prepared CTAC coated gold nanorods using an established protocol.^29^ The resulting nanorods were characterized using scanning transmission electron microscopy (STEM) and UV-vis-NIR spectroscopy (Fig. 2), illustrating that the nanorods are highly monodisperse and produce UV-vis signatures in excellent agreement with previous reports.^29^

**Fig. 2:**
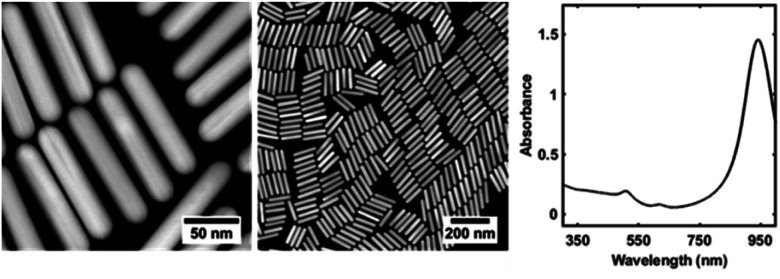
Characterization of gold nanorods. Scanning transmission electron microscopy (STEM) images (left) and UV-vis-NIR spectrum (right) of CTAC coated gold nanorods. Additional characterization of gold nanorods is provided in the ESI.†

Then, we hypothesized that a modified version of the Camden and Jenkins^28,30^ method may produce NHC decorated gold nanorods. We treated aqueous suspensions of nanorods with NHC gold(i) or silver(i) complexes in dichloromethane. The nanorods were characterized using surface-enhanced Raman spectroscopy (SERS), and the spectral signatures were benchmarked to NHC functionalized gold nanospheres.

The SERS spectrum of NHC functionalized gold nanorods is in excellent agreement with previous reports studying the spectral signatures for an NHC bound to gold nanoparticles with diagnostic peaks at approximately 800 cm^−1^, 1300 cm^−1^ and 1400 cm^−1^ (Fig. 3A).^31–33^ SERS is extraordinarily sensitive to ligand binding, and recent studies from our groups demonstrate that NHC SERS signatures change significantly in response to ligand orientation and binding to the gold surface.^28,31–36^ Therefore, if the NHC ligand were to adopt a different binding motif on the nanorod surface, we would expect to observe significant changes in the SERS spectral signatures. SERS characterization of the NHC treated nanorods produces vibrational signatures that are in excellent agreement with the benchmark NHC–Au system on gold nanospheres (Fig. 3A). Although slight differences are observed in the spectra around 800 cm^−1^, the dominant spectral features for all samples are in excellent qualitative agreement. Therefore, these SERS results suggest that the same surface structure forms in all cases, a chemisorbed NHC ligand.

**Fig. 3:**
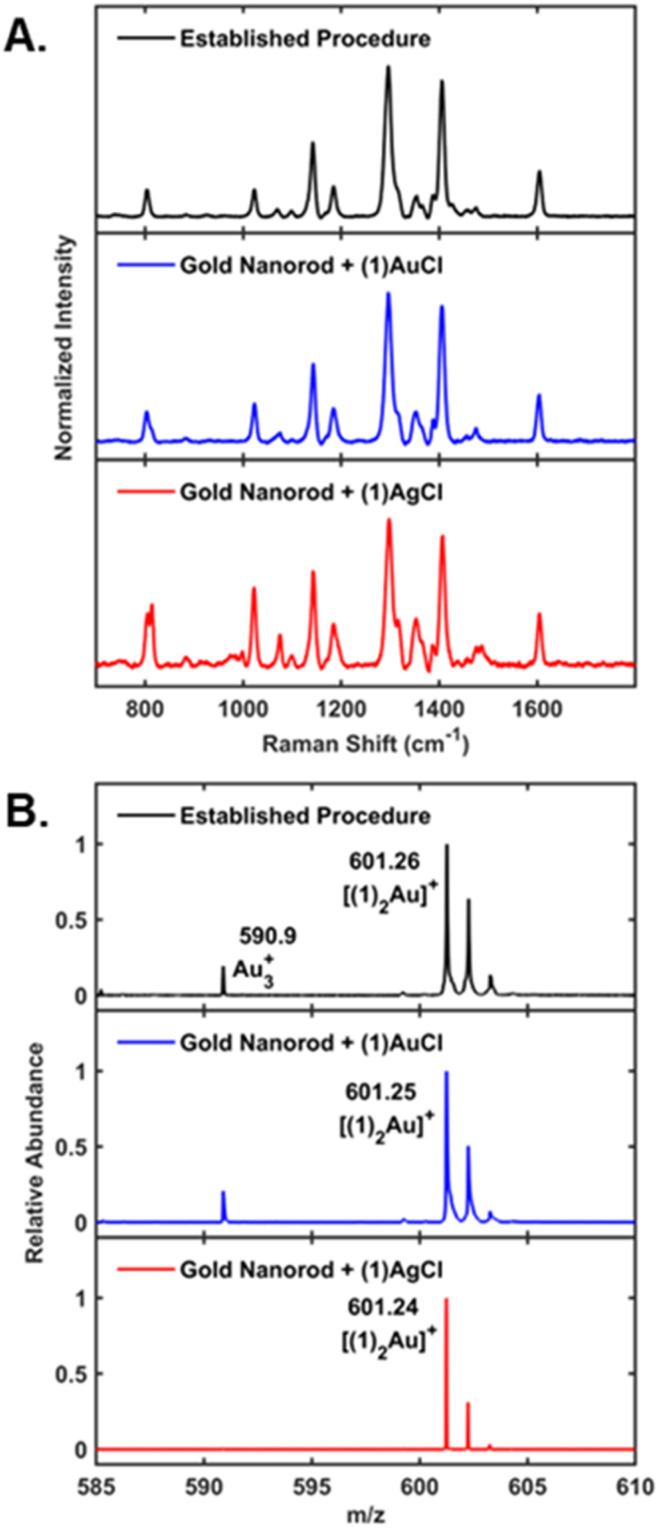
SERS and LDI-MS of NHC nanoparticles. SERS (A) and LDI-MS (B) characterization of standard NHC functionalized gold nanospheres (top, black) and gold nanorods treated with (1)AuCl (middle, blue) or (1)AgCl (bottom, red). The resulting data illustrates that SERS spectroscopic signatures and diagnostic [(1)_2_Au]^+^ ions in LDI-MS are qualitatively indistinguishable for both the established system and the NHC treated nanorods. These data demonstrate that the same surface structure forms in each case, *i.e.* a chemisorbed NHC ligand on gold.

To further explore the NHC binding to the nanorods, we employ laser desorption/ionization mass spectrometry (LDI-MS) and compare our results to the established NHC system (Fig. 3B). All nanoparticles produce [(1)_2_Au]^+^ ions, which are characteristic of NHCs bound to gold.^37–40^ In the case of gold nanorods treated with (1)AgCl, the formation of [(1)_2_Au]^+^ ions illustrates that a transmetalation reaction has occurred to transfer the NHC ligand from a silver atom to a gold atom on the nanorod surface.^30^ Additional characterization and control experiments are given in the ESI.† Overall, these SERS and LDI-MS results illustrate that the NHC complexes transfer the ligand to the gold nanorod surface to form a chemisorbed NHC.

When taken together, we designed a one-step approach to decorate gold nanorods, one of the most widely used classes of nanomaterials, with NHC ligands involving a simple reaction between NHC complexes and CTAC coated gold nanorods. The resulting nanorods were characterized using SERS and LDI-MS to measure the ligand binding, illustrating that the NHC successfully chemisorbs to the nanorod surface. We expect that this procedure will provide a useful starting point for emerging applications of NHC decorated gold nanorods in photothermal therapy and nanorod self-assembly.

## Data availability

The data supporting this study are available from the corresponding authors upon reasonable request.

## Conflicts of interest

There are no conflicts to declare.

## Supplementary Material

RA-015-D5RA00754B-s001
