# Supplementary material for: One-step functionalization of gold nanorods with N-heterocyclic carbene ligands
Source: RSC Adv. 2025 Feb 14;15(7):5007–10. doi: 10.1039/d5ra00754b (PMC11826410; doi:10.1039/d5ra00754b)
Supplement: RA-015-D5RA00754B-s001 [file RA-015-D5RA00754B-s001.pdf]

Supplementary Information (SI) for RSC Advances.
This journal is © The Royal Society of Chemistry 2025

Supplementary Information for "One-Step Functionalization of Gold Nanorods with N-Heterocyclic Carbene Ligands"

Nathaniel L. Dominique,[1] Phattananawee Nalaoh,[2] David M. Jenkins,[2] Richard Vaia,[3] Kyoungweon Park,*[,3,4] Jon P. Camden*[,1]

1. Department of Chemistry and Biochemistry, University of Notre Dame, Notre Dame, Indiana 46556, United States.

2. Department of Chemistry, University of Tennessee, Knoxville, Knoxville, Tennessee 37996, United States.

3. Materials and Manufacturing Directorate, Air Force Research Laboratory, Wright-Patterson AFB, Ohio 45433-7702, United States.

4. Bluehalo, Dayton, Ohio 45432, United States.

*Correspondence to:
Jon P. Camden
Department of Chemistry and Biochemistry
University of Notre Dame
Notre Dame, Indiana 46556
jon.camden@nd.edu

Kyoungweon Park
Materials and Manufacturing Directorate
Air Force Research Laboratory
Wright-Patterson AFB, Ohio 45433-7702
Bluehalo
Dayton, Ohio 45432
Kyoungweon.park@bluehalo.com

Materials and Methods

Materials.

The NHC complex (**1**)AuCl was acquired from STREM chemicals (CAS # 953820-59-2) and used as received. Ultrapure water (>18 megaohms) was prepared in house using a Thermo Fisher Barnstead purification system. Sodium Bromide was purchased from EMD Millipore and dichloromethane was purchased from Macron Fine Chemicals.

Gold Nanorod Functionalization with NHC Au and Ag Complexes.

The functionalization of gold nanorods was performed by adding an aliquot of NHC Au(I) complex or Ag(I) complex to an aqueous suspension of gold nanorods according to modified literature procedures.[1,2] For gold nanorod functionalization with (**1**)AuCl, 10 microliters of a 10 mM (**1**)AuCl stock solution in dichloromethane was added to 1 mL of gold nanorods. Gold nanorods were functionalized with (**1**)AgCl by adding 10 microliters of a 4 mM (**1**)AgCl stock solution in dichloromethane to 1 mL of gold nanorods. The nanoparticle samples were vortex mixed after adding the NHC complexes.

Synthesis of NHC Decorated Gold Nanospheres.

Citrate-capped gold nanoparticles were synthesized according to the Lee and Meisel[3] method and characterized using UV-Vis (Figure S5). Previous reports from our research group demonstrates that this nanoparticle synthesis protocol produces quasi-spherical AuNPs with an average diameter of 23 ± 5 nm.[1,4] Lee and Meisel colloids were functionalized with NHC ligands using the Camden and Jenkins[2] method. Briefly, 1 microliter of 10 mM NHC gold(I) complex in dichloromethane was added per 1 mL of gold colloids and the sample was vortex mixed. These citrate-capped nanoparticles self-aggregated at this concentration.

Surface-Enhanced Raman Spectroscopy (SERS).

All SERS measurements were performed using a custom Raman spectrometer, which has been described elsewhere.[5–8] Briefly, a 633 nm CW HeNe laser (Thor Labs) was directed into an inverted microscope (Nikon Ti-U) and focused onto the nanoparticle sample using a 20x objective (Nikon, NA = 0.50). Scattered radiation was collected using the same objective, filtered through a Rayleigh rejection filter, and directed into a spectrometer (Acton SP2300, 1200 grooves per mm grating). Light was then dispersed onto a liquid nitrogen cooled CCD camera (Princeton Instruments). Data was exported from Winspec software and analyzed in MATLAB. The data was baseline subtracted using the *msbackadj* function in MATLAB and plotted as the average of at least three measurements.

Gold nanorod samples were aggregated for SERS analysis by adding 200 microliters of gold nanoparticles to a vial followed by 160 microliters of sodium bromide (1 M aqueous solution). The samples were vortex mixed and nanoparticle aggregate formation was observed. Gold nanoparticles synthesized according to the Lee and Meisel[3] method self-aggregated after addition of the NHC gold(I) complex and were analyzed without the addition of sodium bromide.

Laser Desorption/Ionization Mass Spectrometry (LDI-MS).

Mass spectrometry measurements were conducted using a Bruker UltrafleXtreme MALDI-ToF instrument at the Notre Dame Mass Spectrometry and Proteomics Facility. This mass spectrometer is equipped with a frequency tripled ND:YAG laser. All measurements were

conducted in positive ion mode with the reflectron in operation. The mass spectrometer was calibrated using gold clusters.[9] In general, the mass spectra were collected by summing together 500 shots for each sample, and either the Small or Ultra beam parameters were used.

UV-Vis Spectroscopy.
Nanoparticles were analyzed using a VWR UV-3100 Spectrophotometer equipped with D2 and tungsten lamps. Disposable plastic cuvettes were used for all measurements. Nanorods and nanospheres were diluted with ultrapure water for UV-Vis Measurements.

Synthesis of CTAC Coated Gold Nanorods.
Chemicals and Materials: Hexadecyltrimethylammonium chloride (CTAC, ≥ 99 %) was purchased from TCI america. Hexadecyltrimethylammonium bromide (CTAB, ≥ 99%) was purchased from GFS chemicals. $HAuCl_4$ (≥ 99.995 %), sodium borohydride, ascorbic acid, and citric acid (≥ 99 %) were purchased from Aldrich and used without further purification.

Methods: Au seeds were prepared in 2 steps which consists of the synthesis of initial seeds by fast reduction of gold precursor and the annealing process. The initial seeds were prepared in 10 mL aqueous CTAC (50 mM) solution containing $HAuCl_4$ (0.25 mM) and citric acid (5 mM). Upon addition of freshly prepared $NaBH_4$ (0.6 mL, 10 mM) under vigorous stirring at room temperature, the mixture turned from light yellow to brown indicating the formation of gold nanoparticles less than 2 nm. Stirring continued for two minutes and the seed solution was covered and heated in an oil bath at 85 °C for 4 hours under gentle stirring, to induce twin formation. During the annealing process, the color of the solution changed from brown to red, indicating the increase in size. The annealed seed solution was stored at room temperature. AuNRs were grown via seed mediated method by adding the annealed seeds to the growth solution. The growth solution was formulated by mixing CTAC, CTAB, and $HAuCl_4$ aqueous solution (bulk concentration of 100 mM, 2mM, and 0.5 mM respectively). 10 mL of growth solution was incubated in a water bath at 25°C for 30 minutes under gentle stirring. Ascorbic acid was added (0.75 mM) which changes the solution color from yellow to clear. Immediately after, a predetermined volume (250 to 500 μL) of the seed solution was combined with the growth solution under gentle stirring. The mixture was left for 30 minutes and subjected to two rounds of centrifugation (21300 rcf for 15 minutes each) to remove excess surfactants and redispersed in CTAC 0.5 mM aqueous solution ([AuNR]~0.5nM).
The morphology and average size of the nanoparticles were characterized through STEM (Scanning Transmission Electron Microscopy) analysis, conducted using an FEI Talos microscope operating at 200 kV. Over 500 particles were measured for each sample to determine the average size and size distribution.

Synthesis of (1)AgCl.
Silver nitrate (1.10 g, 6.47 mmol, 1.5 eqv.) was dissolved in water (10 mL). Then, KOH solution (10.0 g KOH in 50 mL H_2O) was added and stirred at room temperature for 2 hours. The black suspension of Ag_2O was formed, then separated by filtration, washed with cold deionized water (10 mL), and used without further purification.
(1)(Cl) (1.00 g, 4.19 mmol, 1 eqv.), which was prepared using the previously reported anion-exchange resin method,[10] was dissolved in 25 mL CH_2Cl_2. Then, Ag_2O was added and stirred at room temperature. After 2 hours, the reaction mixture was filtered through Celite pad, washed

with CH_2Cl_2 (30 mL × 3 times), and concentrated to dryness. The crude product was redissolved in 5 mL CH_2Cl_2 and triturated with diethyl ether (50 mL). The solid was collected by filtration and dried in the air to obtain white solid powder of (**1**)AgCl as a desired product. The spectroscopic data correlated to the previously reported (**1**)AgCl.[10]

Yield: 890 mg, 62%.

^{1}H NMR (500 MHz, DMSO-d$_6$): δ 7.94 (dd, *J* = 6.2, 3.2 Hz, 2H), 7.43 (dd, *J* = 6.2, 3.1 Hz, 2H), 5.10 (hept, *J* = 6.9 Hz, 2H), 1.68 (d, *J* = 6.9 Hz, 12H).
^{13}C{^{1}H} NMR (126 MHz, DMSO-d$_6$): δ 133.05, 124.25, 113.31, 52.94, 22.88.
MS (DART) *m/z*: [M - Cl]$^+$ Calcd for $C_{13}H_{18}AgN_2$ 309.0448; Found 309.0516.

Characterization data for (1)(AgCl)

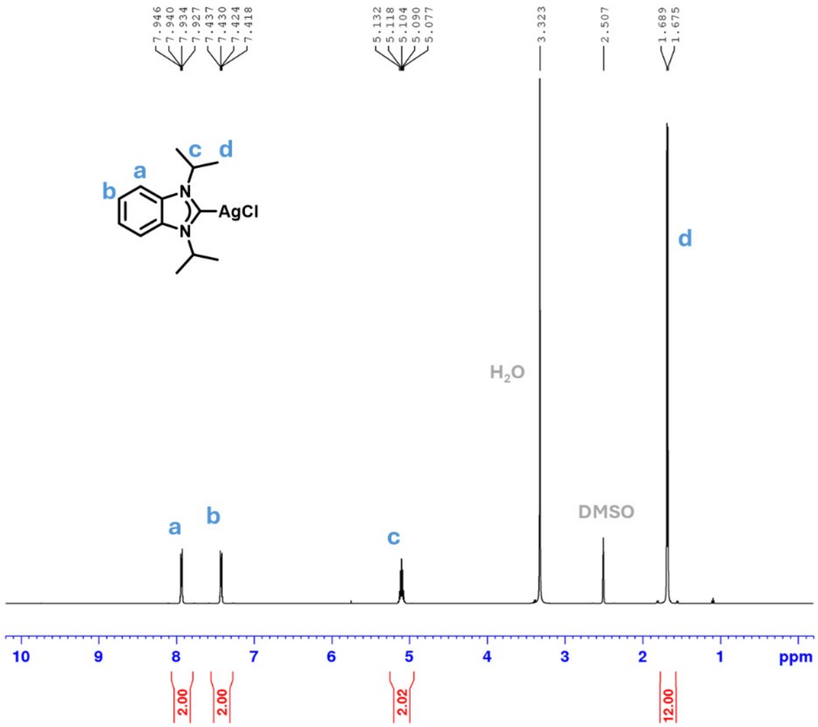

Figure S1. ^{1}H NMR spectrum of **(1)(AgCl)**

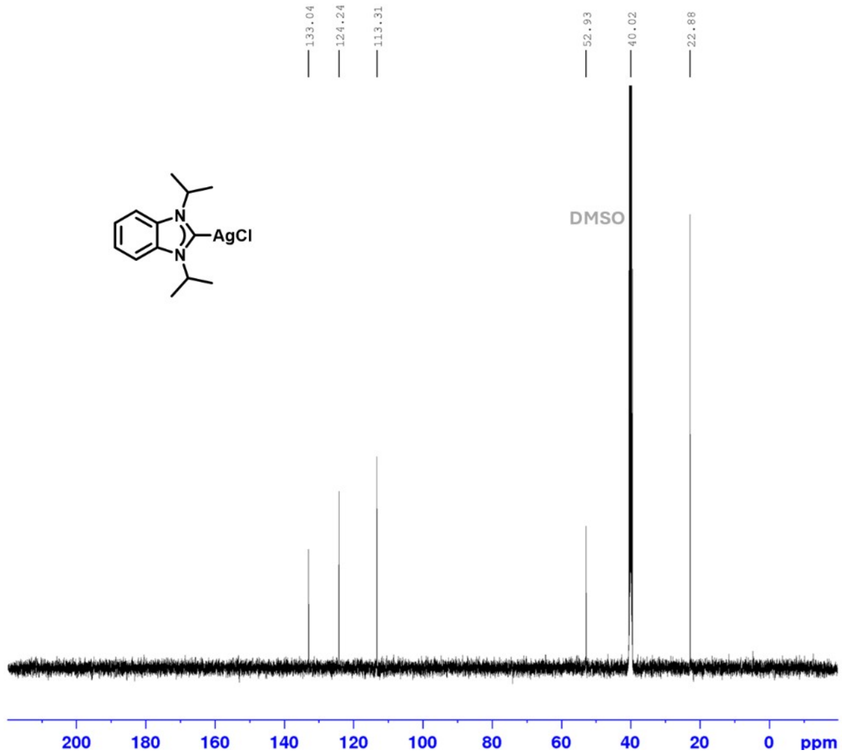

Figure S2. ^{13}C NMR spectrum of **(1)(AgCl)**

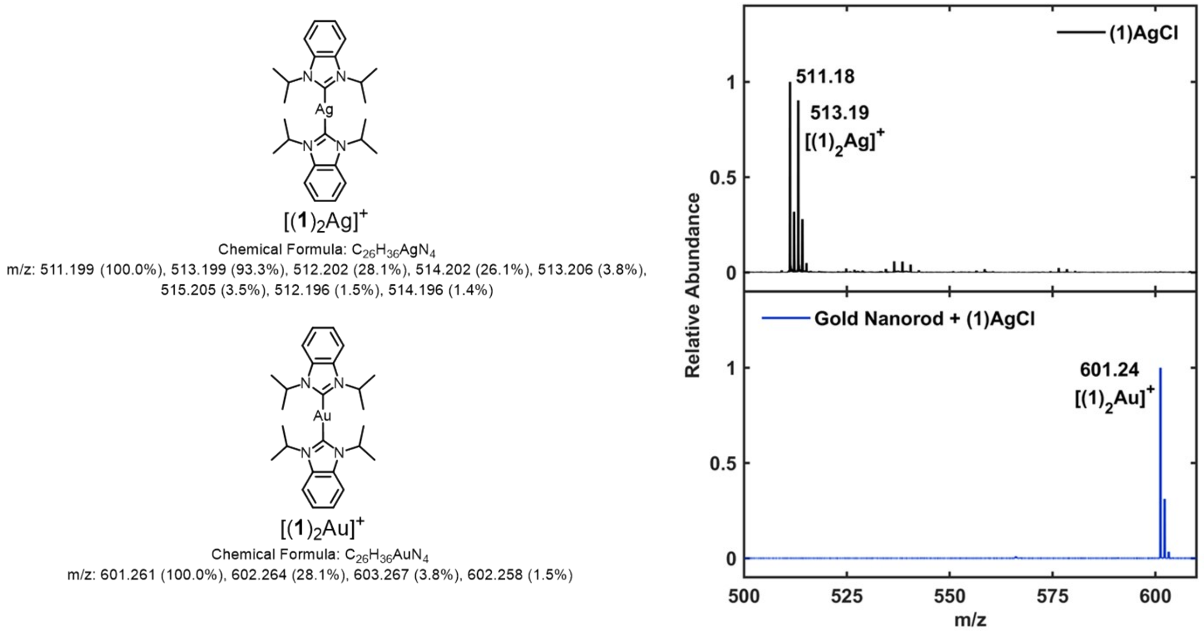

Figure S3. Ions observed in LDI-MS (left). LDI-MS spectra for (**1**)AgCl complex (right, top) and gold nanorods treated with (**1**)AgCl (right, bottom). The LDI-MS spectrum for (**1**)AgCl was calibrated using silver clusters.

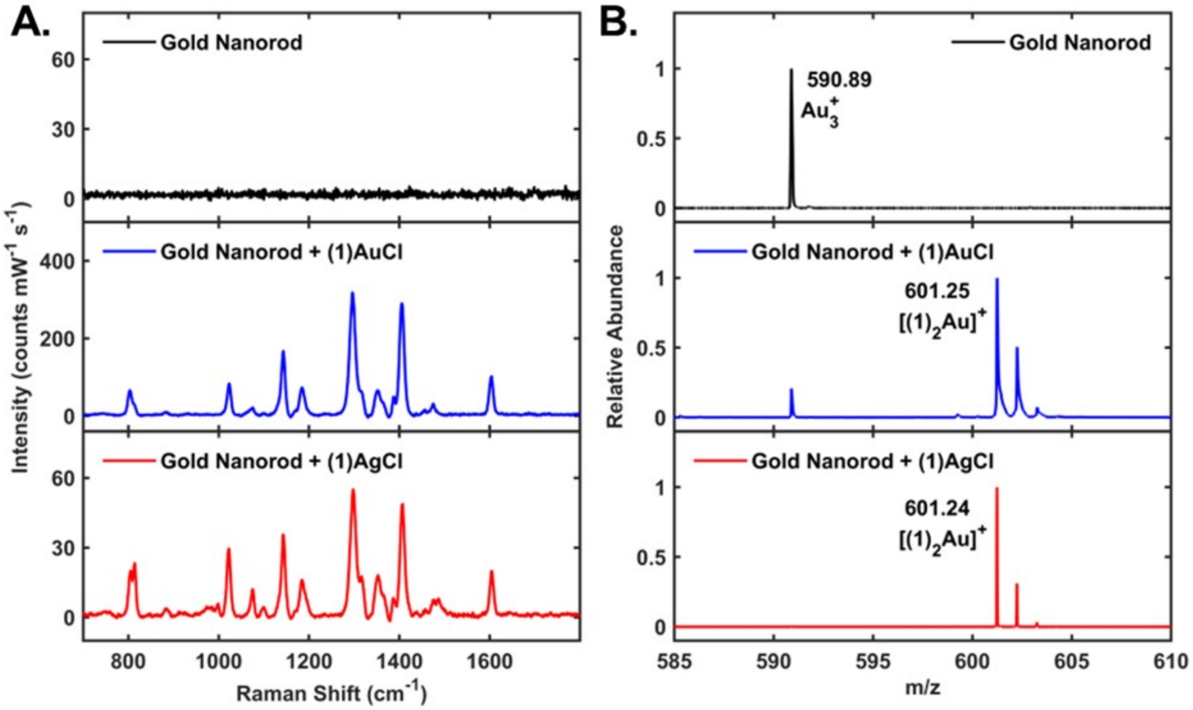

Figure S4. SERS (**A**) and LDI-MS (**B**) of gold nanorods (top), nanorods treated with (**1**)AuCl (middle), and nanorods treated with (**1**)AgCl (bottom). The $[(\mathbf{1})_2Au]^+$ ions in LDI-MS and peaks at approximately 800 cm^{-1}, 1300 cm^{-1}, and 1400 cm^{-1} in SERS – which are characteristic of an NHC bound to gold[1,4,11–15] – were not observed for the control gold nanorod sample.

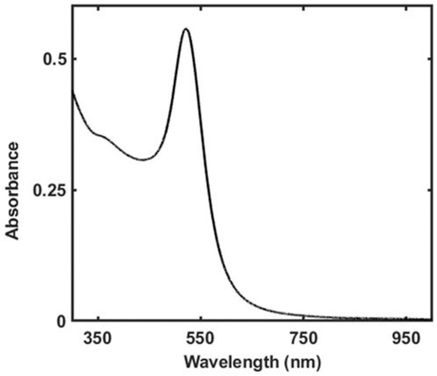

Figure S5. UV-Vis spectrum of gold nanoparticles synthesized according to the Lee and Meisel[3] method.

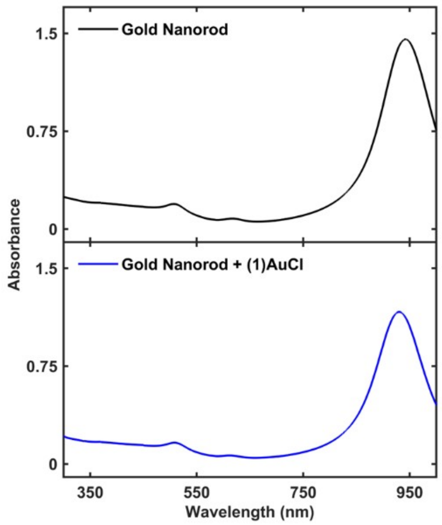

Figure S6. UV-Vis spectrum of gold nanorods (top) and gold nanorods treated with (**1**)AuCl.

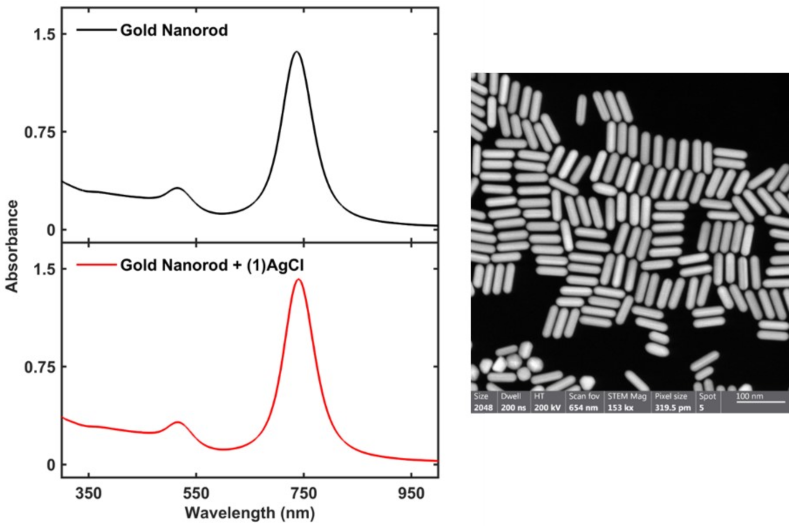

Figure S7. UV-Vis spectrum of gold nanorods (top) and gold nanorods treated with (**1**)AgCl (bottom). STEM image of gold nanorods used for (**1**)AgCl experiments (right).

References

(1) Dominique, N. L.; Chen, R.; Santos, A. V. B.; Strausser, S. L.; Rauch, T.; Kotseos, C. Q.; Boggess, W. C.; Jensen, L.; Jenkins, D. M.; Camden, J. P. Ad Aurum: Tunable Transfer of N-Heterocyclic Carbene Complexes to Gold Surfaces. *Inorg Chem Front* **2022**, *9* (23), 6279–6287. https://doi.org/10.1039/d2qi01941h.

(2) DeJesus, J. F.; Sherman, L. M.; Yohannan, D. J.; Becca, J. C.; Strausser, S. L.; Karger, L. F. P.; Jensen, L.; Jenkins, D. M.; Camden, J. P. A Benchtop Method for Appending Protic Functional Groups to N-Heterocyclic Carbene Protected Gold Nanoparticles. *Angewandte Chemie International Edition* **2020**, *59* (19), 7585–7590. https://doi.org/10.1002/anie.202001440.

(3) Lee, P. C.; Meisel, D. Adsorption and Surface-Enhanced Raman of Dyes on Silver and Gold Sols. *Journal of Physical Chemistry* **1982**, *86* (17), 3391–3395. https://doi.org/10.1021/j100214a025.

(4) Dominique, N. L.; Jensen, I. M.; Kaur, G.; Kotseos, C. Q.; Boggess, W. C.; Jenkins, D. M.; Camden, J. P. Giving Gold Wings: Ultrabright and Fragmentation Free Mass Spectrometry Reporters for Barcoding, Bioconjugation Monitoring, and Data Storage. *Angewandte Chemie International Edition* **2023**, *62* (21), e202219182. https://doi.org/10.1002/anie.202219182.

(5) Preston, A. S.; Hughes, R. A.; Dominique, N. L.; Camden, J. P.; Neretina, S. Stabilization of Plasmonic Silver Nanostructures with Ultrathin Oxide Coatings Formed Using Atomic Layer Deposition. *The Journal of Physical Chemistry C* **2021**, *125* (31), 17212–17220. https://doi.org/10.1021/acs.jpcc.1c04599.

(6) Demille, T. B.; Hughes, R. A.; Dominique, N.; Olson, J. E.; Rouvimov, S.; Camden, J. P.; Neretina, S. Large-Area Periodic Arrays of Gold Nanostars Derived from HEPES-, DMF-, and Ascorbic-Acid-Driven Syntheses. *Nanoscale* **2020**, *12* (31), 16489–16500. https://doi.org/10.1039/d0nr04141f.

(7) Chen, B.-A.; Dominique, N. L.; Kipkorir, A.; Camden, J. P.; Ptasinska, S.; Kamat, P. V. From Light to Dark: Dancing with Electrons in Colloidal 2D MoS2 Nanosheets. *J Phys Chem Lett* **2024**, *15* (18), 4920–4927. https://doi.org/10.1021/acs.jpclett.4c00454.

(8) Lawson, Z. R.; Preston, A. S.; Korsa, M. T.; Dominique, N. L.; Tuff, W. J.; Sutter, E.; Camden, J. P.; Adam, J.; Hughes, R. A.; Neretina, S. Plasmonic Gold Trimers and Dimers with Air-Filled Nanogaps. *ACS Appl Mater Interfaces* **2022**, *14* (24), 28186–28198. https://doi.org/10.1021/acsami.2c04800.

(9) Kolarova, L.; Prokes, L.; Kucera, L.; Hampl, A.; Pena-Mendez, E.; Vanhara, P.; Havel, J. Clusters of Monoisotopic Elements for Calibration in (TOF) Mass Spectrometry. *J Am Soc Mass Spectrom* **2017**, *28* (3), 419–427. https://doi.org/10.1007/s13361-016-1567-x.

(10) Gil-Moles, M.; O'Beirne, C.; Esarev, I. V; Lippmann, P.; Tacke, M.; Cinatl, J.; Bojkova, D.; Ott, I. Silver N-Heterocyclic Carbene Complexes Are Potent Uncompetitive Inhibitors of the Papain-like Protease with Antiviral Activity against SARS-CoV-2. *RSC Med Chem* **2023**, *14* (7), 1260–1271. https://doi.org/10.1039/D3MD00067B.

(11) DeJesus, J. F.; Trujillo, M. J.; Camden, J. P.; Jenkins, D. M. N-Heterocyclic Carbenes as a Robust Platform for Surface-Enhanced Raman Spectroscopy. *J Am Chem Soc* **2018**, *140* (4), 1247–1250. https://doi.org/10.1021/jacs.7b12779.

(12) Trujillo, M. J.; Strausser, S. L.; Becca, J. C.; DeJesus, J. F.; Jensen, L.; Jenkins, D. M.; Camden, J. P. Using SERS To Understand the Binding of N-Heterocyclic Carbenes to

Gold Surfaces. *J Phys Chem Lett* **2018**, *9* (23), 6779–6785. https://doi.org/10.1021/acs.jpclett.8b02764.

(13) Dominique, N. L.; Chandran, A.; Jensen, I. M.; Jenkins, D. M.; Camden, J. P. Unmasking the Electrochemical Stability of N-Heterocyclic Carbene Monolayers on Gold. *Chemistry - A European Journal* **2023**. https://doi.org/10.1002/chem.202303681.

(14) Dominique, N. L.; Strausser, S. L.; Olson, J. E.; Boggess, W. C.; Jenkins, D. M.; Camden, J. P. Probing N-Heterocyclic Carbene Surfaces with Laser Desorption Ionization Mass Spectrometry. *Anal Chem* **2021**, *93* (40). https://doi.org/10.1021/acs.analchem.1c02401.

(15) Kaur, G.; Dominique, N. L.; Hu, G.; Nalaoh, P.; Thimes, R. L.; Strausser, S. L.; Jensen, L.; Camden, J. P.; Jenkins, D. M. Reactivity Variance between Stereoisomers of Saturated N-Heterocyclic Carbenes on Gold Surfaces. *Inorg Chem Front* **2023**, *10* (21). https://doi.org/10.1039/d3qi01541f.
